# Understanding Differential Stress and Mental Health Reactions to COVID-19-Related Events

**DOI:** 10.3390/ijerph20105819

**Published:** 2023-05-13

**Authors:** Rita Sebastião, David Dias Neto, Vasco Costa

**Affiliations:** 1 School of Psychology, ISPA—Instituto Universitário, 1149-041 Lisbon, Portugal; dneto@ispa.pt (D.D.N.); 26054@alunos.ispa.pt (V.C.); 2 APPsyCI—Applied Psychology Research Center Capabilities & Inclusion, ISPA—Instituto Universitário, 1149-041 Lisbon, Portugal

**Keywords:** COVID-19, COVID-19-related events, negative mental health, positive mental health, stress responses

## Abstract

The effects of the pandemic on mental health can be studied through different variables, such as the number of COVID-19 stressors, the stressor types, and the stress responses. Understanding the sources of mental strain is crucial for developing effective interventions. The present study analyzed the relationship between these COVID-19-related variables and positive and negative mental health. A cross-sectional study was conducted with 666 individuals from the Portuguese general population, mostly females (65.5%) between 16–93 years old. They completed self-report measures regarding the number of COVID-19 stressors, the stressor types, the stress responses (IES-R), and positive (MHC-SF) and negative mental health (BSI-18). The results demonstrated that a higher number of COVID-19-experienced stressors and more stress responses were related to worse mental health. Regarding stressor types, experiences not related to the COVID-19 infection (e.g., tension at home) presented the largest effects on mental health. The strongest predictor was the stress responses for negative (*β* = 0.50) and positive mental health (*β* = −0.17). The predictors explained more about negative mental health than positive. These findings support the idea that individual appraisals play a crucial role in mental health.

## 1. Introduction

The COVID-19 pandemic has triggered new challenges for the population’s mental health and worsened existing mental health difficulties [1,2,3,4,5]. Since the start of the COVID-19 pandemic, this effect has been studied from different perspectives. According to most stress process models [6], stress is composed of environmental demands (the stressors) and the appraisal of an event as harmful or threatening (stress perceptions/responses). The literature occasionally focuses on stressors and at other times on the cognitive, emotional, and biological responses such stressors evoke. These models suggest that the degree to which an individual experiences stress is affected by both the objective characteristics of the stressors and the individual appraisal of these stressors [7]. It is essential to distinguish between perceptions of stress and exposure to stressors when studying the links between stress and health [7]. Stressors and stress responses have been associated with mental health [8,9,10].

According to this idea, the COVID-19 pandemic has been studied through different variables that addressed exposure to stressors and stress responses. For instance, research has considered the number of COVID-19 stressors [11], the COVID-19 stressor types (e.g., lockdown periods, being infected) [12], and the COVID-19 stress responses [13]. Based on that, the effects of the pandemic on mental health can be considered based on these different variables. Although they may all be important predictors of mental health [6,7,14], their differential roles in mental health has not been studied.

The two-continua model of mental health suggests that mental health is the combination of positive and negative mental health, or well-being and ill-being [15,16]. Positive mental health or well-being encompasses the existence of satisfaction in emotional, social, and psychological aspects [16]. Negative mental health or ill-being includes a range of struggles, such as depression and anxiety [17]. A comprehensive understanding of mental health is attainable by simultaneously measuring well-being and these difficulties. The absence of mental illness does not entirely reflect the presence of well-being or vice versa [16]. Additionally, despite their intercorrelation, these two factors are distinct and may act relatively independently [15,16] and as such should both be considered in research on mental health, providing greater insights into this topic [18]. Nevertheless, almost no research integrated positive and negative mental health during the COVID-19 pandemic. Studying both outcomes in this context is important because the predictors of negative mental health may not necessarily be the opposite of those associated with positive mental health [19]. 

Concerning the number of COVID-19 stressors, adverse mental health consequences can occur if exposure to stressors occurs repeatedly, if it is not self-limited, and if the individual does not adapt to a repeated stressor. Within COVID-19 research, results show that individuals who experienced more COVID-19 stressors demonstrated lower well-being levels [11], higher depressive symptoms [1], and higher anxiety and post-traumatic symptoms [20]. Despite this, no studies have compared the effect of the number of COVID-19 stressors on positive and negative mental health. 

Regarding the COVID-19 stressor types, most of the literature developed around the world has focused on discrete COVID-19 stressors (e.g., lockdown) or the pandemic as a general construct, and their impact on mental health [12,21,22,23,24,25,26,27,28,29]. It is equally important to study the full range of COVID-19 stressor types and not reduce the COVID-19 pandemic to discrete stressors or a general concept. The following COVID-19 stressors have been associated with mental health issues: (a) having an acquaintance, close friend, or relative infected with COVID-19 [2]; (b) relatives or close friends being hospitalized due to COVID-19 infection [30,31]; (c) death of relatives or close friends due to COVID-19 [2]; (d) testing positive for COVID-19 [2]; (e) being hospitalized due to COVID-19 infection [32,33]; (f) having long COVID [34]; (g) increased tension (e.g., verbal arguments or conflicts) at home [35,36]; (h) financial and work concerns [37]; (i) worsening of work/study conditions [35]; (j) job loss and financial difficulties [36]; and (k) lockdown, quarantine, and isolation [35]. Kira et al. [38] and Valiente et al. [39] tried to summarize some of these COVID-19 stressors, and found that lockdown was the strongest predictor of post-traumatic stress disorder, depression, and anxiety [38] and that anxiety about COVID-19 was the strongest predictor of negative mental health [39]. The strongest predictor of positive mental health was the absence of economic threat associated with COVID-19 [39]. However, no studies integrated all these COVID-19 stressor types nor analyzed their impact on positive and negative mental health after controlling the effect of co-occurring stressor types.

Finally, how people perceive an event’s impact on their life shapes reactions to the stressor [6,7,40]. Studies on psychological and environmental stressors often focus on stress exposure. However, individual appraisals seem to be a more reliable predictor of mental health outcomes [7,9]. Some studies investigated the impact of stress responses during the COVID-19 pandemic. For example, Yang et al. [41] found that academic workload, separation from school, and fears of contagion contributed to general perceived stress, which, in turn, negatively influenced well-being. Bridgland et al. [42] concluded that the worst event reported related to the pandemic was the strongest predictor of post-traumatic symptoms in comparison with exposure to COVID-19 stressors. On the other hand, no studies have investigated the effect of stress responses on positive and negative mental health. 

The literature suggests that the COVID-19 pandemic’s different variables play an important role in mental health. To further our understanding of the role of these different variables in mental health, it is essential to study their differential impact. Based on this rationale, the present study has two main goals: (1) analyze the relationship between the following COVID-19-related variables—number of experienced stressors, stressor types, and stress responses—and positive and negative mental health; (2) analyze the global effect of these variables on mental health. These might all be important predictors, but according to the literature [6,7,14], it is expected that stress responses will be the strongest predictor of positive and negative mental health in comparison with stress exposure measures, contributing to understanding the stress models and the two-continua model in a crisis context. Additionally, acknowledging the origins of the burden on individuals is essential to understanding who is more vulnerable to the pandemic. This is crucial for developing more effective interventions to reduce psychopathological symptoms and increase well-being, working toward complete mental health.

## 2. Materials and Methods

### 2.1. Design

This research followed a cross-sectional correlational study design in order to test the relationship between COVID-19-related variables and their global effect on mental health. The study focused on three predictor variables: the number of experienced stressors, stressor types, and stress responses related to COVID-19. The criterion variables were positive and negative mental health.

### 2.2. Participants

The study was conducted with a convenience sample of 666 participants from the Portuguese general population. Participants were mostly female, between the ages of 16–93 years, and married/in a civil union or single. Most worked full-time or were retired. Most have a typical Portuguese education level, BA or high school. Right before the pandemic, 11.7% were diagnosed with a mental health disorder. Additionally, 40.7% indicated that they had at least one disease that put them at risk for COVID-19. For a more detailed analysis of the sample’s characteristics, see Table 1 (sample’s characteristics per criterion variables are presented as Supplementary Materials).

### 2.3. Measures

A questionnaire was included to assess the following sociodemographic variables: sex, age, education level, marital status, employment status, prior mental health disorder diagnosis, and physical diseases related to COVID-19 risk factors (e.g., hypertension, heart or lung conditions). Additionally, the following self-report questionnaires were included:

#### 2.3.1. COVID-19-Related Events Characterization

This contains questions created for the present study, based on the literature [43,44,45], that assess the number and types of COVID-19 stressors. Participants were asked to identify all 11 COVID-19 stressors they had experienced since the onset of the pandemic (e.g., “I have tested positive for COVID-19”) and then to indicate the most stressful event they had experienced and the time elapsed since it occurred.

#### 2.3.2. Impact of Event Scale-Revised (IES-R, [46]; Portuguese Version by Vieira et al. [47])

This is a 22-item questionnaire used to evaluate stress responses to a specific event (in this case, it was considered the most stressful COVID-19 event selected before). Items are categorized into four subscales: physiological activation, intrusion, avoidance, and emotional anesthesia. Each item is rated on a five-point Likert scale (1 = never to 5 = always). The current study focuses on the total score, presenting good internal consistency (Cronbach α total score = 0.94).

#### 2.3.3. Brief Symptom Inventory 18 (BSI-18, [48]; Portuguese Version by Canavarro et al. [49])

This consists of 18 items grouped into three symptom scales: somatization, depression, and anxiety. It assesses negative mental health on a five-point Likert scale (0 = not at all to 4 = extremely). The 18-item scores are combined into the global severity index (GSI), a measure of overall levels of psychological distress, with higher scores reflecting more severe psychopathological symptoms [49,50]. The current study focuses on GSI. This measure indicates good internal consistency (Cronbach α GSI = 0.93).

#### 2.3.4. Mental Health Continuum-Short Form (MHC-SF, [51]; Portuguese Version by Fonte et al. [52])

This includes 14 items and evaluates positive mental health. Participants rate each item on a 6-point scale (1 = never to 6 = every day). The items measure the levels of emotional, social, and psychological well-being. The MHC-SF produces an overall score, with higher scores indicating greater well-being. In our sample, Cronbach’s alpha was 0.91 for the overall score.

### 2.4. Procedure

Data collection for this cross-sectional study was conducted online and at hospitals, senior universities, and patient organizations between January 2022 and July 2022. This period was marked by the lifting of most of the COVID-19 restrictions (e.g., wearing a face mask started to be no longer mandatory during this period) in Portugal [53]. The online survey was conducted through Qualtrics and was distributed via social networks. Data collection followed these two approaches (paper- and web-based questionnaires) to include people who might not otherwise have access to the questionnaire, ensuring a diverse sample in terms of stressors and contexts.

Before data collection began, a pilot study was conducted by presenting the questionnaire to 6 individuals from the general population to ensure comprehension of the questionnaire. The ethics committee of the ISPA—Instituto Universitário (D-049-2-22/Jan 2022) and the other organizations in which data were collected approved this study. Informed consent was obtained from all participants in both formats.

### 2.5. Statistical Analysis

Statistical analyses were performed using SPSS [54] and the significance level considered was 0.05. To examine the differences in sociodemographic variables for positive and negative mental health, and to control them in the hierarchical multiple regression analyses, independent samples *t*-tests and Pearson correlations were used.

As were included 11 COVID-19 stressors, we performed an exploratory factor analysis based on the Cramer V correlation matrix to reduce the data to a smaller set of summary variables. This allowed for the analysis of the different types of COVID-19 stressors. The principal component factor analysis method was used for extraction, followed by varimax rotation. Variables included in the factors were those saturated above 0.40 [55]. 

Several hierarchical multiple regression analyses were conducted with positive and negative mental health as criterion variables. Relevant sociodemographic variables were included in the first step. Separate analyses were conducted for the number of experienced stressors, stressor types, and stress responses related to COVID-19. These predictor variables were included in the second step. In the regression, including stress responses, the time elapsed since the event was included in step 1. To examine the specificity of each stressor type’s association with mental health after controlling the effect of co-occurring stressor types, all stressor types were introduced in step 2. VIF was used to evaluate multicollinearity. Finally, to test the moderation effect of stressor type on the relationship between stress responses and mental health, simple moderation analyses were conducted using the PROCESS macro-Model 1 for SPSS [56], one for each criterion variable.

## 3. Results

### 3.1. Sociodemographic Variables and Mental Health

Women, *t*(660) = −3.68, *p* < 0.001, *d* = −0.30, 95% CI [−0.46, −0.14], and those who belonged to the risk group because of physical illness, *t*(663) = −1.80, *p* = 0.036, *d* = −0.14, 95% CI [−0.30, 0.01], reported more psychopathological symptoms. Those who reported mental health issues immediately before the pandemic reported less well-being, *t*(640) = 5.36, *p* < 0.001, *d* = 0.66, 95% CI [0.41, 0.90], and more psychopathological symptoms, *t*(639) = −8.48, *p* < 0.001, *d* = −1.04, 95% CI [−1.29, −0.79]. Finally, age was positively correlated with well-being (*r* = 0.14, *p* < 0.001) and negatively with psychopathological symptoms (*r* = −0.13, *p* < 0.001). These sociodemographic variables will be controlled in the following analyses. 

### 3.2. Number of COVID-19 Stressors

The participants reported, on average, three stressors (0–11 stressors, *SD* = 1.97) related to COVID-19. The analysis investigating the cumulative effect of COVID-19-related stressors on negative and positive mental health after entering control variables indicated that the models accounted for 22.5% of the variance of negative mental health, *F*(5, 630) = 37.84, *p* < 0.001. Additionally, it accounted for 7.3% of the variance of positive mental health, *F*(5, 635) = 17.66, *p* < 0.001. The number of COVID-19-related stressors was a significant predictor of negative mental health (*β* = 0.30, b = 2.22, 95% CI [1.71, 2.73], *t* = 8.59, *p* < 0.001) and of positive mental health (*β* = −0.10, b = −0.70, 95% CI [−1.21, −0.18], *t* = −2.65, *p* = 0.008).

### 3.3. COVID-19 Stressors Frequency and COVID-19 Stressor Types

Table 2 provides an overview of participants’ exposure frequency to each COVID-19 stressor. As were included 11 COVID-19 stressors, an exploratory factor analysis was performed to reduce data to a smaller set of summary variables for subsequent analyses. Four factors/COVID-19 stressor types were identified: (1) own experiences related to the COVID-19 infection, including the following variables—testing positive for COVID-19, being hospitalized due to COVID-19 infection, and having long COVID; (2) experiences not (directly) related to the COVID-19 infection, including the following variables—increased tension at home (e.g., verbal arguments or conflicts), job loss and financial difficulties, worsening of work/study conditions, and increase in responsibilities as a caregiver; (3) loved ones’ serious experiences related to the COVID-19 infection, including the following variables—relatives/close friends being hospitalized due to COVID-19 infection, and death of relatives/close friends due to COVID-19; (4) loved ones’ COVID-19 positive test and isolation/quarantine, including the following variables—isolation/quarantine, relatives/close friends testing positive for COVID-19.

### 3.4. COVID-19 Stressor Types

Analyses evaluating the specificity of each experienced COVID-19 stressor type’s association with negative mental health, after controlling the effect of co-occurring stressor types and sociodemographic variables, are presented in Table 3. This table shows that, when controlling for sociodemographic variables and the cooccurrence of all stressor types, only own experiences related to the COVID-19 infection and experiences not (directly) related to the COVID-19 infection were statistically significant. After entering control variables (demographics and co-occurring stressor types), the model accounted for 23.9% of the variance of negative mental health, *F*(8, 627) = 25.91, *p* < 0.001.

Analyses assessing the specificity of each COVID-19 stressor type’s association with positive mental health after controlling the effect of co-occurring stressor types and sociodemographic variables are presented in Table 4. As seen in this table, results indicated that no differences were observed when controlling for sociodemographic variables and co-occurring stressor types. After entering control variables (demographics and co-occurring stressor types), the model accounted for 8.2% of the variance of positive mental health, *F*(6, 632) = 10.46, *p* < 0.001.

### 3.5. Stress Responses to the Most stressful Experienced COVID-19-Related Event

The most common event type selected as most stressful was loved ones’ COVID-19 positive test and isolation/quarantine (38.3%, *n* = 241), followed by experiences not (directly) related to the COVID-19 infection (25.6%, *n* = 161). Concerning the stress responses to the most stressful event (*M* = 52.59, *SD* = 17.31), participants reported a lower mean than in the adaptation study of the scale for the Portuguese population in the community sample [47]. The time elapsed since the event, reported by 611 individuals, varied between 0 and 28 months (*M* = 9.09, *SD* = 7.45). 

The analysis investigating the effect of the stress responses to the most stressful experience on negative mental health, after controlling relevant variables, indicated that the model accounted for 36.2% of the variance in negative mental health, *F*(6, 562) = 54.62, *p* < 0.001. Furthermore, the stress responses to the most stressful event were a significant predictor. The same analysis was performed on positive mental health, but the model accounted only for 9.8% of the variance, *F*(4, 567) = 16.54, *p* < 0.001, even though the stress responses to the most stressful experience were a significant predictor of well-being after entering control variables (Table 5).

Regarding the moderation effect of the stressor type, the models did not improve when the interactions were included (negative mental health: *R*^2^ change = 0.0014, *F*(3, 597) = 0.40, *p* = 0.753, positive mental health: *R*^2^ change = 0.0016, *F*(3, 597) = 0.33, *p* = 0.803). No interactions were observed for the stressor type, indicating that the stressor type does not moderate the association between the stress responses to the most stressful event and mental health.

## 4. Discussion

Concerning the first goal of our study, of analyzing the effect of the following COVID-19-related variables—number of experienced stressors, stressor types, and stress responses—on mental health, we found that a higher number of COVID-19-experienced stressors was related to more psychopathological symptoms and less well-being, as expected [11,20]. 

Results showed a specificity of each COVID-19 stressor type association with negative mental health. After controlling demographic variables, the largest effect sizes found were for experiences not (directly) related to the COVID-19 infection, followed by own experiences related to the COVID-19 infection. This differed from other studies, such as Kira et al. [38] study, conducted with a Turkish sample, which found that disrupted routines and isolation (vs. fears and economic issues) presented the largest effect. Nevertheless, these studies did not include events related to the consequences of COVID-19 for the person and their loved ones. Similar to our results, Alzueta et al. [35] found that the greatest effects on negative mental health came from economic issues, work-related issues, and social aspects, such as tension at home. Regarding positive mental health, the largest effect size was experiences not (directly) related to the COVID-19 infection. This result was consistent with Valiente et al. [39] study, which found that the strongest predictor of positive mental health was the absence of economic threat associated with COVID-19.

Despite these results, it is possible that some of the observed associations for a given COVID-19 stressor type were secondary to the cooccurrence of another COVID-19 stressor type. When the analysis was controlled for other COVID-19 stressor types, for example, the loved one’s serious experiences related to the COVID-19 infection type was no longer significant in the case of negative mental health. The loss of significance suggests that its association with negative mental health was secondary to the occurrence of some other stressor types. In comparison, results for positive mental health suggest that, after controlling for the cooccurrence of other COVID-19 stressor types, the stressors continue to be significantly associated with positive mental health. This indicates that, for these stressors, the association with positive mental health was not the result of co-occurring stressor types. It was expected that different COVID-19 stressor types could differently impact mental health. However, there is not enough COVID-19 literature investigating this matter. It can be observed that despite the differences, the strongest predictor of positive and negative mental health is the experiences not (directly) related to the COVID-19 infection. In other words, non-life-threatening stressors seem to have a greater impact on mental health, although more research is necessary given the small effect sizes. Additionally, it is essential to test these models in other contexts.

Regarding the stress responses to the most stressful event on mental health, as expected [41,42], more stress responses were related to more psychopathological symptoms and lower levels of well-being. The absence of a moderator effect of the COVID-19 stressor type suggests that stress responses are more important than the COVID-19 stressor type. Furthermore, diathesis-stress models suggest that individual characteristics, such as psychological processes, may play a significant role in how stressful events contribute to mental health issues. Therefore, it would be beneficial for future studies to incorporate the role of psychological processes in these models. 

Concerning the second aim of our study of analyzing the global effect of these variables on mental health, the stress responses to the most stressful event were the strongest predictor of mental health compared to the rest of the predictors. This result is consistent with the idea that individual appraisals are reliable predictors of mental health outcomes [6,7,9], and is consistent with Bridgland et al.’s [42] study on post-traumatic stress disorder symptomology. The results contributed to understanding the two-continua model of mental health in the context of a health crisis. According to this model, different predictors can have a different effect on each of these outcomes. The number of COVID-19-experienced stressors, stressor types, and stress responses to the most stressful event explained more negative mental health than positive. This can occur because the predictors are negative ones and do not include positive predictors. Future studies should address this issue. 

Nevertheless, the results should be interpreted considering some limitations. These limitations include the cross-sectional design of this study, which constrains conclusions about causality. The present study indicates an association between variables, allowing the exploration of a new situation and giving clues about relevant variables. However, follow-up data are being collected. The convenience sampling method used in this study may limit the generalizability and representativeness of the results. However, the main concern of the present research was to have a relevant number of participants per event, rather than to have national representativeness. Additionally, using self-report questionnaires to collect data simultaneously introduces a common method bias that could affect the results. Furthermore, stressors were recalled retrospectively, which could introduce recall bias. Multiple comparisons were conducted. However, our study is exploratory to future analyses intended to determine which stressor factors are important contributors to mental health. Alpha-level adjustments reduce the likelihood of making a type I error, but the probability of making a type II error increases. Additionally, in an exploratory study, following this approach can mean potentially meaningful findings are missed. Presenting all the results without alpha-level adjustments allow us to present a complete picture of our study, and for most of the results the *p*-value is <0.001. Finally, it should be noted that the data were collected when most COVID-19 restrictions had been lifted [53]. There was more knowledge about the virus, including the availability of vaccines, which could have influenced the outcomes. 

Regardless of the limitations, the current research has significant implications. The present study summarizes the significant impact of COVID-19-related events on mental health. There was a significant impact of the number of experienced stressors and COVID-19 stressor types. However, the stress responses to the most stressful event, which depend on the individual’s evaluation, appear to be the strongest predictor of mental health outcomes. This is consistent with the idea that individual appraisals play a crucial role in mental health [7]. Additionally, the present research allows for studying the stressor and the stress it causes. This contributes to the understanding that the number of experienced stressors or COVID-19 stressor types alone does not allow us to fully explain the relevant information. Another strength of the current study, in a period marked by ongoing COVID-19 stressors of different types, is that we controlled for other types when we analyzed the COVID-19 stressor type. Therefore, we can estimate the impact and effect size of different COVID-19 stressors on mental health. Concerning the COVID-19 stressor type, the present study analyzes the different stressors, contrary to most studies focusing on discrete COVID-19 stressors (e.g., lockdown) or the pandemic as a general concept. Finally, comparing negative and positive mental health, which few studies have done concerning the pandemic, contributes to the two-continua model of mental health, which suggests an intercorrelation between positive and negative mental health. However, this relationship may vary in certain aspects [15,16]. Clinically, the results highlighted the diversity of direct, indirect, and non-life-threatening stressors that impact mental health and the essential role of individual appraisals on mental health. To date, few studies have compared the impact of specific pandemic-related stressors or patterns of stressors on functioning. This is necessary to understand the origins of burden in individuals and develop more efficient interventions for present and future crises. This and similar studies contribute to developing resilience in the face of challenges. Our results highlight the importance of addressing stress appraisals in psychological interventions.

## 5. Conclusions

In conclusion, we found an increase in negative mental health and a decrease in positive mental health due to different COVID-19-related variables, even in a period marked by the lifting of most of the COVID-19 restrictions and more knowledge about the virus. Notably, many predictors (number of experienced stressors, COVID-19 stressor types, and stress responses to the most stressful event) predicted both outcomes. However, the strongest predictor was stress responses to the most stressful event. This study plays a role in enhancing the understanding of stress models. Additionally, the results contribute to understanding the two-continua model of mental health in the context of a health crisis. Despite the common predictors, the models presented some differences. In general, the predictors contributed to explaining more of the negative mental health effects than the positive.

Furthermore, when considering the specificity of each COVID-19 stressor type’s association with mental health, the results suggest that personal events are more important to positive mental health than events occurring with others. If future studies confirm these results, they will allow us to understand the origins of the burden in individuals toward developing more efficient interventions to decrease psychopathological symptoms and increase well-being. 

## Figures and Tables

**Table 1:** Sample Characterization.

Sample Demographics Characteristic	Cases (*n* = 666)
Female	436 (65.8%)
Age (mean [*SD*])	47.15 (20.26)
Marital status	
Single	255 (38.4%)
Married/in a civil union	306 (46.1%)
Divorced	66 (9.9%)
Widowed	37 (5.6%)
Level of education	
Four years or less	27 (4.1%)
Six years	9 (1.4%)
Nine years	54 (8.1%)
Twelve years	193 (29%)
Bachelor’s degree	261 (39.2%)
Master’s degree	109 (16.4%)
Ph.D. or higher	13 (2%)
Professional status	
Full-time employee	282 (42.3%)
Part-time worker	31 (4.7%)
Unemployed	48 (7.2%)
Student	96 (14.4%)
Retired	209 (31.4%)
Mental health disorders diagnosed	75 (11.7%)
Belonging risk group	271 (40.7%)

Note. Variable distributions are reported as *n* (%) unless otherwise specified.

**Table 2:** Frequencies of COVID-19 stressors.

COVID-19 Stressor	% Participants Experienced the Stressor
Test positive for COVID-19	38.3
Being hospitalized due to COVID-19 infection	11.9
Long COVID	16.7
Relatives/close friends tested positive for COVID-19	73.3
Relatives/close friends being hospitalized due to COVID-19 infection	19.2
Death of relatives/close friends due to COVID-19	12.9
Isolation/quarantine	39.5
Job loss and financial difficulties	12
Increase of the responsibilities as a caregiver	20.3
Increase tension at home	16.2
Worsening of work/study conditions	31.8

**Table 3:** Regression analyses of the stressor type on negative mental health.

	Results for Each COVID-19 Stressor Type								Results for Each COVID-19 Stressor Type Considering the Cooccurrence of All COVID-19 Stressor Types						
		95%CI for *b*								95%CI for *b*					
COVID-19 Stressor Type	*b*	LL	UL	*SE*	*β*	*t*	*p*	*R2a*	*b*	LL	UL	*SE*	*β*	*t*	*p*
Own Infection Experiences	2.81	1.73	3.89	0.55	0.19	5.10	<0.001	0.17	2.20	1.12	3.28	0.55	0.15	3.99	<0.001
Experiences not COVID-19 Infection	4.31	3.23	5.39	0.55	0.28	7.82	<0.001	0.21	3.92	2.84	5.00	0.55	0.26	7.13	<0.001
Loved Ones Serious Experiences	3.08	1.38	4.78	0.87	0.13	3.55	<0.001	0.15	1.65	−0.01	3.31	0.84	0.07	1.96	0.051
Loved Ones’ Positives and quarantine	2.10	0.66	3.53	0.73	0.11	2.86	0.004	0.15	0.53	−0.90	1.96	0.73	0.03	0.73	0.467

Note. Own infection experiences = own experiences related to the COVID-19 infection; experiences not COVID-19 infection = experiences not (directly) related to the COVID-19 infection; loved ones serious experiences = loved ones serious experiences related to the COVID-19 infection; loved ones’ positives and quarantine = loved ones’ COVID-19 positive test and isolation/quarantine.

**Table 4:** Regression analyses of the stressor type on positive mental health.

	Results for Each COVID-19 Stressor Type								Results for Each COVID-19 Stressor Type Considering the Cooccurrence of All COVID-19 Stressor Types						
		95%CI for *b*								95%CI for *b*					
COVID-19 Stressor Type	*b*	LL	UL	*SE*	*β*	*t*	*p*	*R2a*	*b*	LL	UL	*SE*	*β*	*t*	*p*
Own Infection Experiences	−1.39	−2.45	−0.33	0.54	−0.099	−2.58	0.010	0.07	−1.44	−2.54	−0.33	0.56	−0.10	−2.55	0.011
Experiences not COVID-19 Infection	−1.69	−2.78	−0.60	0.56	−0.12	−3.05	0.002	0.08	−1.67	−2.77	−0.57	0.56	−0.12	−2.97	0.003
Loved Ones Serious Experiences	0.21	−1.47	1.89	0.85	0.009	0.25	0.807	0.06	0.79	−0.92	2.49	0.87	0.04	0.91	0.366
Loved Ones’ Positives and quarantine	−0.05	−1.46	1.36	0.72	−0.003	−0.07	0.942	0.06	0.61	−0.86	2.08	0.75	0.03	0.82	0.412

Note. Own infection experiences = own experiences related to the COVID-19 infection; experiences not COVID-19 infection = experiences not (directly) related to the COVID-19 infection; loved ones serious experiences = loved ones serious experiences related to the COVID-19 infection; loved ones’ positives and quarantine = loved ones’ COVID-19 positive test and isolation/quarantine.

**Table 5:** Regression analyses of the stress responses to COVID-19-related events on mental health.

		95% CI for *b*					
	*b*	LL	UL	*SE*	*β*	*t*	*p*
Negative Mental Health Model							
Time elapsed since the event	0.05	−0.08	0.18	0.07	0.03	0.73	0.468
Stress Responses to COVID-19-related event	0.40	0.35	0.46	0.03	0.50	13.85	<0.001
Positive Mental Health Model							
Time elapsed since the event	−0.16	−0.31	−0.02	0.07	−0.09	−2.21	0.028
Stress Responses to COVID-19-related event	−0.13	−0.20	−0.07	0.03	−0.17	−4.14	<0.001

## Data Availability

The datasets generated during and analyzed during the current study are available from the corresponding author upon reasonable request.

## Supplementary Materials

The following supporting information can be downloaded at: https://www.mdpi.com/article/10.3390/ijerph20105819/s1, Table S1. Characteristics of the sample stratified by the COVID-19 stressor types; Table S2. Characteristics of the sample stratified by the number of experienced COVID-19 stressors and stress responses to the most stressful experienced COVID-19-related event.
