# Supplementary material for: Understanding Differential Stress and Mental Health Reactions to COVID-19-Related Events
Source: Int J Environ Res Public Health. 2023 May 13;20(10):5819. doi: 10.3390/ijerph20105819 (PMC10217821; doi:10.3390/ijerph20105819)
Supplement: Supplementary file 1 [file ijerph-20-05819-s001.zip › ijerph-2279110-supplementary.pdf]

Supplementary Materials

Table S1. Characteristics of the sample stratified by the COVID-19 stressor types

	Own Infection Experiences		Experiences not COVID-19 Infection		Loved Ones Serious Experiences		Loved Ones' Positives and quarantine	
Sample demographics characteristic	Yes	No	Yes	No	Yes	No	Yes	No
Female	164 (61.2%)	272 (68.9%)	223 (64.8%)	213 (66.8%)	105 (64.8%)	331 (66.1%)	337 (64.1%)	99 (72.3%)
Age (mean [*SD*])	45.76 (18.46)	48.09 (21.35)	42.84 (18.66)	51.77 (20.90)	51.16 (19.93)	45.84 (20.21)	46.89 (20.60)	48.16 (18.90)
Marital status								
Single	98 (36.4%)	157 (39.7%)	148 (42.9%)	107 (33.5%)	48 (29.3%)	207 (41.4%)	210 (39.8%)	45 (32.8%)
Married/ in a civil union	140 (52%)	166 (42%)	155 (44.9%)	151 (47.3%)	85 (51.8%)	221 (44.2%)	239 (45.4%)	67 (48.9%)
Divorced	20 (7.4%)	46 (11.6%)	32 (9.3%)	34 (10.7%)	20 (12.2%)	46 (9.2%)	48 (9.1%)	18 (13.1%)
Widowed	11 (4.1%)	26 (6.6%)	10 (2.9%)	27 (8.5%)	11 (6.7%)	26 (5.2%)	30 (5.7%)	7 (5.1%)
Level of education								
Four years or less	14 (5.2%)	13 (3.3%)	10 (2.9%)	17 (5.3%)	11 (6.7%)	16 (3.2%)	22 (4.2%)	5 (3.6%)
Six years	5 (1.9%)	4 (1%)	5 (1.4%)	4 (1.2%)	5 (3%)	4 (0.8%)	9 (1.7%)	0
Nine years	30 (11.2%)	24 (6%)	27 (7.8%)	27 (8.4%)	12 (7.3%)	42 (8.4%)	40 (7.6%)	14 (10.2%)
Twelve years	78 (29%)	115 (29%)	106 (30.7%)	87 (27.1%)	52 (31.7%)	141 (28.1%)	153 (28.9%)	40 (29.2%)
Bachelor's degree	94 (34.9%)	167 (42.1%)	129 (37.4%)	132 (41.1%)	58 (35.4%)	203 (40.4%)	198 (37.4%)	63 (46%)
Master's degree	41 (15.2%)	68 (17.1%)	62 (18%)	47 (14.6%)	20 (12.2%)	89 (17.7%)	94 (17.8%)	15 (10.9%)
Ph.D. or superior	7 (2.6%)	6 (1.5%)	6 (1.7%)	7 (2.2%)	6 (3.7%)	7 (1.4%)	13 (2.5%)	0
Professional status								
Full-time employee	148 (55%)	134 (33.8%)	158 (45.8%)	124 (38.6%)	68 (41.5%)	214 (42.6%)	218 (41.2%)	64 (46.7%)
Part-time worker	13 (4.8%)	18 (4.5%)	17 (4.9%)	14 (4.4%)	7 (4.3%)	24 (4.8%)	26 (4.9%)	5 (3.6%)
Unemployed	20 (7.4%)	28 (7.1%)	36 (10.4%)	12 (3.7%)	11 (6.7%)	37 (7.4%)	37 (7%)	11 (8%)
Student	28 (10.4%)	68 (17.1%)	65 (18.8%)	31 (9.7%)	20 (12.2%)	76 (15.1%)	79 (14.9%)	17 (12.4%)
Retired	60 (22.3%)	149 (37.5%)	69 (20%)	140 (43.6%)	58 (35.4%)	151 (30.1%)	169 (31.9%)	40 (29.2%)
Mental health disorders diagnosed	34 (13%)	41 (10.8%)	36 (10.9%)	39 (12.5%)	18 (11.3%)	57 (11.8%)	59 (11.5%)	16 (12.3%)
Belonging risk group	113 (42%)	158 (39.8%)	130 (37.7%)	141 (43.9%)	79 (48.2%)	192 (38.2%)	217 (41%)	54 (39.4%)

Table S2. Characteristics of the sample stratified by the number of experienced COVID-19 stressors and stress responses to the most stressful experienced COVID-19-related event

Variable	Number of experienced COVID-19 stressors	Stress responses to the most stressful COVID-19-related event
Sex		
Male	3.16 (2.13)	49.50 (16.82)
Female	2.79 (1.87)	53.77 (17.43)
Age	*r* = -0.06	r = -0,05
Marital status		
Single	2.85 (1.79)	51.66 (16.60)
Married/ in a civil union	3.10 (2.07)	52.22 (17.23)
Divorced	2.61 (2.07)	56.05 (18.54)
Widowed	2.57 (2.05)	50.85 (21.33)
Level of education		
Four years or less	3.63 (2.00)	63.12 (14.30)
Six years	4.67 (2.55)	65.00 (18.29)
Nine years	3.50 (2.68)	61.62 (18.15)
Twelve years	3.05 (2.24)	54.05 (17.45)
Bachelor's degree	2.62 (1.61)	51.12 (16.46)
Master's degree	2.71 (1.60)	45.32 (16.21)
PhD or superior	3.62 (1.80)	48.69 (13.78)
Professional status		
Full-time employee	3.17 (2.06)	53.30 (17.17)
Part-time worker	3.45 (2.03)	54.87 (16.18)
Unemployed	3.48 (2.25)	59.36 (18.10)
Student	2.84 (1.57)	51.67 (15.65)
Retired	2.41 (1.83)	48.66 (17.71)
Mental health disorders		
Yes	3.12 (2.16)	60.89 (19.20)
No	2.91 (1.95)	51.21 (16.81)
Risk group		
Yes	3.16 (2.22)	54.00 (18.63)
No	2.76 (1.77)	51.20 (16.34)
